# Distribution, Levels, and Risk Assessment of Polycyclic Aromatic Hydrocarbons (PAHs) in Some Water Bodies along the Coastal Belt of Ghana

**DOI:** 10.1100/tsw.2010.96

**Published:** 2010-06-01

**Authors:** David Kofi Essumang

**Affiliations:** Department of Chemistry, University of Cape Coast, Cape Coast, Ghana

**Keywords:** polycyclic aromatic hydrocarbons, risk assessment, Pra Estuary, Sakumono lagoon, Benya lagoon, Narkwa lagoon

## Abstract

The levels and distribution of 24 polycyclic aromatic hydrocarbons (PAHs) were determined in six water bodies along the coastal belt of Ghana using gas chromatography with flame-ionization detection (GC/FID). The average total PAHs recorded are from the Pra estuary, 6.3 μg/L; Benya lagoon, 7.5 μg/L; Sakumono lagoon, 10.1 μg/L; lower Volta estuary, 26.3 μg/L; Keta lagoon, 10.6 μg/L; and Narkwa lagoon, 16.1 μg/L.The 12 PAHs that were well distributed in all the coastal waters analyzed include naphthalene, pyrene, fluorene, 2-methylnaphthalene, 2,6-dimethylnaphthalene, acephnaphthalene, acephnaphthene, 1-methylphenanthrene, 2,3,5-trimethylnaphthalene, chrysene, biphenyl, and phenanthrene. The presence of benzo(b)fluoranthene, benzo(a)anthracene, and benzo(j,k)fluoranthene in some of the water bodies is a source of concern as they have been classified by the International Agency for Research on Cancer (IARC) and U.S. Environmental Protection Agency (EPA) as probable human carcinogens. These water bodies are used for fishing and for some domestic purposes by the people living around them, thereby exposing them to some dangers and the risk of getting cancer. The human health cancer risk assessment carried out also indicates that there is the possibility of some users of the water bodies getting cancer in their lifetime.

## INTRODUCTION

Polycyclic aromatic hydrocarbons (PAHs), a group of stable chemical compounds including two or more rings, are ubiquitous organic contaminants (xenobiotics) in the environment and belong to the group of persistent organic pollutant (POPs). Sixteen have been identified as priority pollutants by the U.S. Environmental Protection Agency (EPA) and the European Union (EU)[1]. They have relatively low solubility in water, but are highly lipophilic. PAHs bind with ash and move long distances through the air, especially those that are lighter, before those that are soluble get dissolved in rainwater and/or into rivers and groundwater sources[2,3]. When dissolved in water or adsorbed to particulate matter, PAHs can undergo photodecomposition when exposed to ultraviolet light from solar radiation[4,5].

PAHs are hazardous as they increase the risk of cancer[6,7], and create advanced glycogen end products, which leads to an increased risk of coronary heart disease and diabetes[8]. Some people who have inhaled or have been in contact with mixtures of PAHs and other chemicals for long periods of time have developed cancer[9]. These compounds are also bioaccumulative and may cause a number of health-related complications, ranging from mutations in lower animals to cancerous cells in humans[10,11].

It is generally accepted that virtually all the PAHs emitted into the atmosphere (natural and anthropogenic) are associated with aerosols, soot, and other airborne particulate matter, which transport PAH over intermediate and long distances. Because of their hydrophobic properties, PAHs in aquatic environments also rapidly become associated to particulate matter. Hence, particle transport is important for the fate of PAHs in marine and estuarine environments[12].

The coastline of Ghana, which forms part of the Gulf of Guinea, consists of about 90 lagoons and other water bodies[13]. There are two main types of lagoons: “open” and “closed”. The former are always open to the sea and are associated with rivers that flow all year round, while the latter remain closed to the sea for most or part of the year[13,14,15]. The quality of both types is often influenced by human activities inland, and the water bodies serve as traps for materials brought from the inland areas as a result of flocculation, precipitation, and sedimentation, or by careless direct waste disposal into the lagoons. Most of these water bodies have become sinks for most domestic and municipal waste[16]. Also, most of the lagoons and rivers pass under bridges of heavily trafficked roads before joining the sea, thereby receiving sizeable amounts of emissions from the traffic, including PAHs. Urban run-off contaminated with PAHs may be an environmental threat to water resources as some levels of PAHs have been found in roadside soils in Ghana[17].

In view of the toxic nature of PAHs, the knowledge of the sources and fate of these compounds in the environment is very important. Industrial activities, fossil fuel combustion, vehicle washing, open dumps[16], and solid waste burning (all of which currently occur in the vicinity of most of these water bodies) are known anthropogenic sources of PAHs[18,19]. However, there are few scientific data on the levels and distribution of PAHs in the marine environment in Ghana[20,21].

Meanwhile, PAHs have received considerable attention as environmental organic pollutants in many continents, such as North America, Europe, and Asia. A great number of PAHs have been identified and quantified in these countries in virtually all segments of the environment due to their possible health effects at very low concentrations[22]. This area of research needs considerable attention in Ghana because studies carried out[17,20,21] show the presence of PAHs in the Ghanaian environment. This study investigated the PAH levels and distribution along the coastal zone (lagoons and some estuaries) along the Gulf of Guinea in some selected communities in Ghana. The results have been used to quantify the potential toxicological effect of PAHs in these water bodies as some of them are used extensively for fishing and domestic purposes.

## MATERIALS AND METHODS

### Sampling Sites

The coastal belt of Ghana is densely populated due to the intensive fishing activities that take place there. Most of the activities at all the sampling sites are very homogeneous, except for Sakumono lagoon (D)(Fig. 1), which receives effluents from an oil refinery and an aluminum facility. The lower Volta (E) is the estuary where the biggest manmade lake in Ghana enters the sea. The lake also houses the turbines that provide hydroelectric power to Ghana. The water bodies have a number of agricultural activities along their banks and there are several solid-waste disposal sites scattered along the banks as well, with occasional burning in an attempt to reduce the waste size. There are a number of two-stroke engine outboard motors operating on these water bodies that tend to leak oil into the water[23,24] and coal tar is

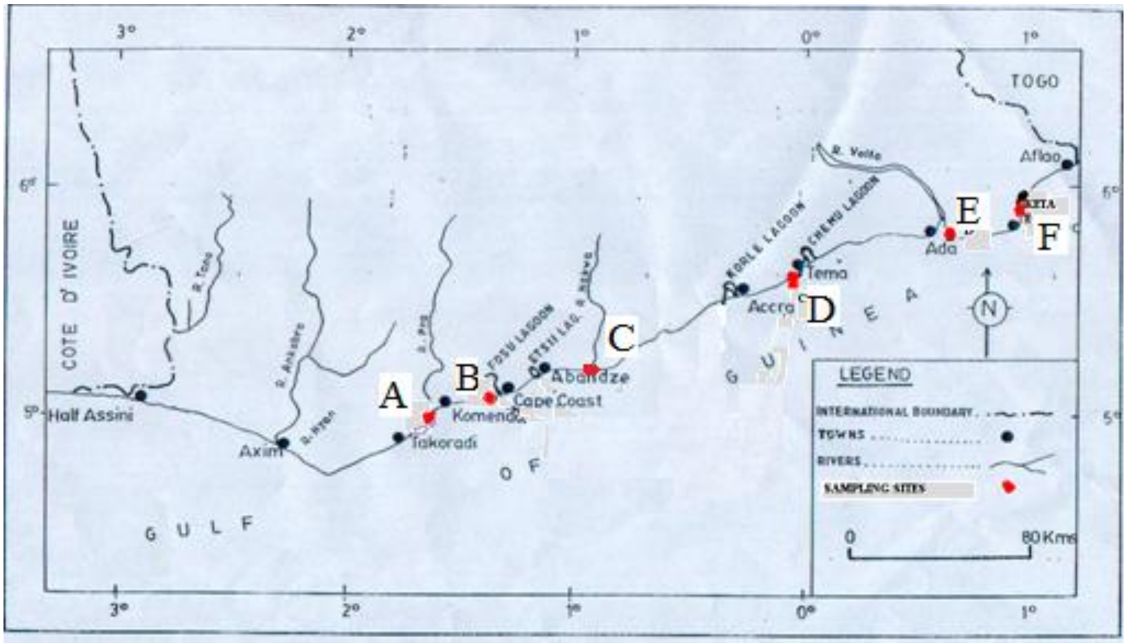

**FIGURE 1**. Map of southern Ghana showing the sample sites. A = Pra estuary, B = Benya lagoon, C = Narkwa lagoon, D = Sakumono lagoon, E = lower Volta, F = Keta lagoon.

usually used to paint the fishing vessels in order to protect them from destruction. In addition, most of these water bodies pass under heavily trafficked bridges before entering into the sea. All of these activities may have an influence on the distribution of PAHs in these water bodies[25].

## Sample Collection

Samples (composite from three points on each water body) of the water were collected ten times between January and April 2007 from all six sampling sites. This period in Ghana is the dry season with no or very little rain. In all, ten samples were collected from each sampling site for the analysis. Clean amber glass bottles were used in the collection of the samples to prevent degradation from solar irradiation and bacteria growth[26]. The amber glass bottles were washed with detergents (liquid soap) and rinsed with a lot of water to remove any trace of the soap. Distilled water was then used to wash the bottles to remove ions present. The samples were carefully collected directly by hand (with gloves) into sampling bottles by pressing them under the water surface to avoid the surface layer, where surfactive materials may accumulate. The sampling bottles were twice partially filled, shaken well, and emptied before the actual filling was done. The filled bottles were immediately covered, properly labeled, and placed in an ice chest with ice for further treatment and analysis in the laboratory.

## Extraction of PAH from Water

About 1.0 L of filtered (Whatman's filter paper of pore size 0.45 μm, Cellulose Nitrate) water sample (composite) from each site was poured into a separating funnel; 50 mL of dichloromethane was added followed by 0.2 mL internal standard. The content of the separating funnel was shaken well for the dichloromethane to extract as much organic components as possible from the water sample. The separating funnel was left undisturbed on a retort stand for some time so that the mixture separated into the organic and water layers. The organic layer was drained through a glass funnel, which was plugged with glass wool, filter paper, and sodium sulfate into a Zymark tube. The sodium sulfate was used to absorb water that might still be present in the organic layer.

A second extraction was carried out using an additional 50 mL of dichloromethane using the same procedure as described above, and the extract was added to the first one in the Zymark tube. One drop of iso-octane was added to the content in the Zymark tube and the tube was placed into a Turbo Evaporation Unit to reduce the volume to 1 mL. The iso-octane served as a keeper to prevent evaporation of the needed components. The extract in the Zymark tube was then transferred into a test tube using Pasteur pipette. The Zymark tube was washed with 2 mL of dichloromethane and added to the content in the test tube. The test tube was heated in a block heater and a gentle stream of nitrogen gas was used to reduce the volume to 0.5 mL. About 1.0 mL of cyclohexane was added and the mixture was evaporated to dryness followed by the addition of 0.5 mL hexane[27,28].

## Clean-Up

Most of the unwanted components were removed from the extract, leaving the components of interest. This was achieved by using solid-phase extraction tubes containing 500 mg florisil, 3 mL by volume. This solid phase was conditioned using 6 mL of hexane; 0.5 mL of the extract was added and eluted with 6.0 mL hexane into a test tube. The PAHs in the extract were kept by the florisil column. The column was eluted again using 20% dichloromethane in hexane into another test tube and this fraction contained the PAHs. The volume was reduced to 0.5 mL and was transferred into sample vials for gas chromatography with flame-ionization detection (GC/FID)[9,29].

## Quality Assurance and Quality Control

Recovery studies were done that involved the use of PAH-certified reference materials from the National Institute of Standards and Technology (SP 1, NIST – 194B, USA). This certified sample has 26 different standard PAHs. The certified reference sample was subjected to the same extraction procedure as applied for all the water samples. Recoveries were calculated from the differences in PAH-certified concentrations and the concentrations obtained after analysis using GC/FID.

Results from the NIST reference material showed high recovery of PAHs, ranging from 94 to 101%, with an average PAH recovery of 98%. The results from the NIST reference material indicate reliability of the extraction system as well as the efficiency of the GC/FID instrument. In fact, the use of the soil reference material to assess PAH extraction efficiency from the water matrix is debatable since the two samples are of two different matrices. However, the results were used to establish the reliability of the extraction system as well as the elution efficiency of the GC/FID instrument since there was no certified reference material for the sample matrix under study at the time of the analysis.

## Calculation of Carcinogenic Risk

Human health evaluation computerized software-RISC 4.02[30,31] was used in the evaluation of the cancer and noncancer risk assessment. Carcinogenic risks are estimated as the incremental probability of an individual developing cancer over a lifetime as a result of exposure to the potential carcinogen. This risk is referred to as the individual excess lifetime cancer risk (IELCR) or just carcinogenic risk. Published values of chemical carcinogenic toxicity (slope factor) are used to calculate risk from the Lifetime Average Daily Dose (LADD):

$$IELCRij = SFij\ LADDij \tag{1}$$

Where IELCRij = individual excess lifetime cancer risk for chemical i exposure route i[dimensionless], SFij = slope factor for chemical i exposure route j[mg/kg-d]-1, LADDij = lifetime average daily dose for chemical i exposure route j[mg/kg-d].

## RESULT AND DISCUSSION

The results from this study showed elevated levels of some PAHs of the various water bodies (Table 1). However, the levels obtained were low as compared to those from other studies[20,21,32,33,34]. This may be due to the fact that all the water bodies in the present study flow into the sea (open lagoons), thereby reducing the chemical load.

**TABLE 1**
**The Average PAH Concentrations (n = 10) of the Water Samples**

| Types PAHs | Concentration in µg/L | | | | | |
|---|---|---|---|---|---|---|
| | A | B | C | D | E | F |
| Naphthalene | 0.1 | nd | 0.2 | 0.2 | 0.8 | 0.2 |
| 2-Methylnaphthalene | 1.3 | 1.7 | 4.1 | 2.7 | 0.4 | 3.0 |
| 1-Methylnaphthalene | nd | nd | 0.2 | nd | nd | nd |
| 2,6-Dimethylnaphthalene | 0.3 | 0.1 | 0.2 | 1.1 | 0.4 | 0.5 |
| Biphenyl | 0.1 | nd | 0.3 | nd | 1.3 | 0.2 |
| Acephnaphthalene | 2.9 | 1.6 | 1.2 | 1.3 | 3.5 | 1.0 |
| Acephnaphthene | 0.1 | 0.6 | 2.5 | 1.0 | 5.6 | 1.6 |
| 2,3,5-Trimethylnaphthalene | 0.1 | 0.5 | 0.5 | 0.3 | 1.4 | 0.2 |
| Fluorene | 0.1 | nd | nd | 0.1 | 1.3 | 0.2 |
| Phenanthrene | 0.1 | nd | 0.03 | 0.05 | 0.4 | nd |
| Anthracene | 0.3 | nd | nd | 0.6 | 1.2 | nd |
| 1-Methylphenanthrene | 0.8 | 0.4 | 1.9 | 0.6 | 4.7 | 1.5 |
| Fluoranthene | nd | nd | nd | 0.01 | nd | 0.2 |
| Pyrene | 0.03 | 2.2 | 1.1 | 0.02 | 1.3 | 0.3 |
| Benzo(a)anthracene | nd | nd | nd | nd | 0.3 | nd |
| Chrysene | nd | nd | nd | 1.4 | 3.7 | 1.2 |
| Benzo(b)fluoranthene | nd | 0.4 | nd | 0.7 | nd | nd |
| Benzo(j,k)fluoranthene | 0.1 | nd | 3.9 | nd | nd | 0.5 |
| Benzo(e)pyrene | nd | nd | nd | nd | nd | nd |
| Benzo(a)pyrene | nd | nd | nd | nd | nd | nd |
| Perelyne | nd | nd | nd | nd | nd | nd |
| Indeno(1,2,3,cd)pyrene | nd | nd | nd | nd | nd | nd |
| Dibenz(a,h)anthracene | nd | nd | nd | nd | nd | nd |
| Benzo(g,h,i)pyrelene | nd | nd | nd | nd | nd | nd |
| Total PAHs | 6.3 | 7.5 | 16.1 | 10.1 | 26.3 | 10.6 |

*Note:* A = Pra estuary, B = Benya lagoon, C = Narkwa lagoon, D = Sakumono lagoon, E = lower Volta estuary, F = Keta lagoon. nd = below detection limit of 0.001 µg/L.

## Distribution of PAHs in the Coastal Waters

Twelve of the PAHs were found in all the coastal waters analyzed and these include naphthalene, pyrene, fluorene, 2-methylnaphthalene, 2,6-dimethylnaphthalene, acephnaphthalene, acephnaphthene, 1-methylphenanthrene, 2,3,5-trimethylnaphthalene, chrysene, biphenyl, and phenanthrene. However, most of the carcinogenic PAHs were below the detection limit (<0.001 µg/L) of GC employed in the analysis.

The distribution of PAHs in this study (Fig. 2) followed a similar pattern as that obtained by Maskaoui et al.[32], Zhou and Maskaoui[33], Wattayakorn[34], and El-Deeb and Emara[35]. This may be a result of indiscriminate dumping and burning of municipal waste, which is a common feature in most of these environments in Ghana, especially at the Narkwa lagoon (C). Also, coal tar is used extensively to coat the canoes (fishing vessels) and most of the two-stroke engines of the outboard motors used are in a poor condition, and hence may have substantial PAH emissions. It could also be as a result of exhaust emissions from vehicles that ply these areas to cart fish. The Keta lagoon also has a number of onion farms around it and any chemical used on them may enter the lagoon. These activities may have influence on the distribution of PAHs in these water bodies[25].

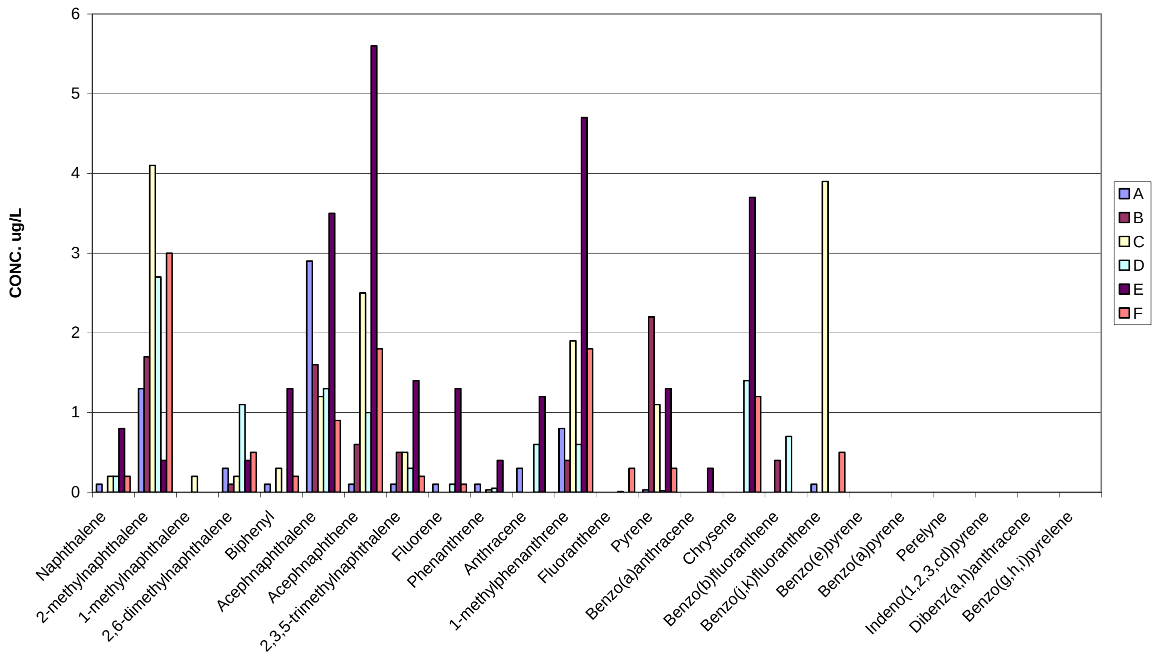

**FIGURE 2.** The average PAHs found in the coastal waters.

Benzo(e)pyrene, benzo(a)pyrene, perelyne, indeno(1,2,3,cd)pyrene, dibenz(a,h)anthracene, benzo(g,h,i)pyrelene, fluoranthene, benzo(a)anthracene, benzo(b)fluoranthene, benzo(j,k)fluoranthene, and chrysene were only scarcely found in all the coastal water samples. However, the presence of benzo(a)anthracene, benzo(b)fluoranthene, and benzo(j,k)fluoranthene in some of the water bodies is a source of worry since they have been stated by the International Agency for Research on Cancer (IARC) and EPA as probable human carcinogens. Most of the heavy-molecular-weight PAHs are well-known carcinogens and, therefore, their absence is good for the environment. It is worth noting that even though the substances are below detection limit, it does not imply that they are completely absent and all effort must be made so that they are not continuously dispersed. These water bodies are used by the people living around them for several human activities, thereby exposing them to several dangers and even the risk of getting cancer[7,10].

## Total PAHs

The average levels of total PAHs recorded (in μg/L) are as follows: Pra estuary, 6.3; Benya lagoon, 7.5; Sakumono lagoon, 10.1; lower Volta estuary, 26.3; Keta lagoon, 10.6; and Narkwa lagoon, 16.1. Hence, the highest level of total PAH was found at the lower Volta estuary, which makes it the most polluted site. This is the estuary of the biggest lake in Ghana running from the north to the south, reflecting inland activities and serving as a trap for materials brought from the inland area as a result of flocculation, precipitation, and sedimentation, or by careless waste disposal directly into the river. There are also hydroelectric power turbines in a dam upstream with a number of electrical transformers and other electrical installations. However, the total levels obtained in this study are generally lower than those reported by Maskaoui et al.[32], Zhou and Maskaoui[33], Wattayakorn[34], Gilbert et al.[20], and Simpson et al.[36], but are comparable to those by Essumang et al.[21].

## PAHs at the Individual Sites

### Pra Estuary

The Pra River passes through very important gold-mining communities in Ghana. Sometimes, some alluvial gold mining occurs in the upper parts of the river. The large-scale mining activities upstream use a great deal of heavy equipment, thereby releasing a high amount of combustion-related particulate matter into the river. The following PAHs were determined: naphthalene, 2-methylnaphthalene, 2,6-dimethylnaphthalene, acephnaphthalene, anthracene, 1-methylphenanthrene, 2,3,5-trimethylnaphthalene, fluorene, acephnaphthene, biphenyl, phenanthrene, pyrene, and benzo(j,k)fluoranthene (Fig. 3). The presence of benzo(j,k)fluoranthene raises concern since it has been classified by the IARC and EPA as a probable human carcinogen. This water body is used for fishing and domestic use by the people living around it, thereby exposing them to several dangers and the risk of getting cancer[10].

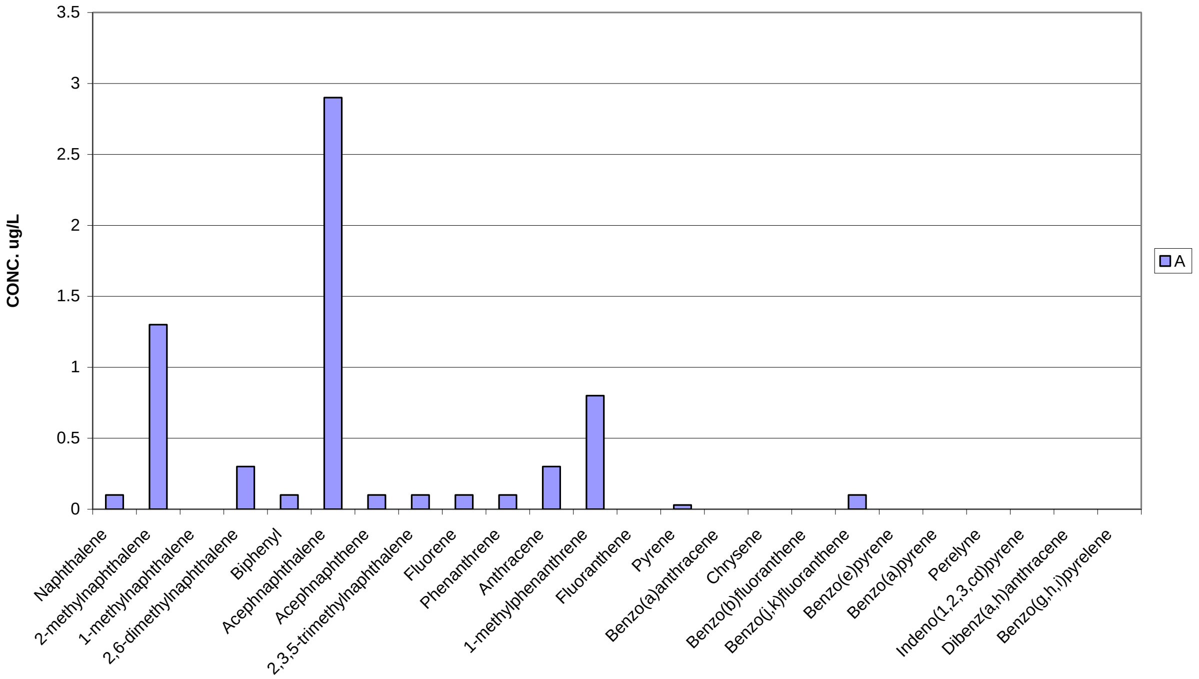

**FIGURE 3.** The average PAHs found in the Pra estuary.

## Benya Lagoon

The fourth largest fishing harbor in Ghana is sited in this lagoon, attracting quite a number of fishing trawlers from all corners of the country. The following PAHs were determined: 2-methylnaphthalene, acephnaphthalene, acephnaphthene, 2,3,5-trimethylnaphthalene, 1-methylphenanthrene, pyrene, benzo(b)fluoranthene, and 2,6-dimethylnaphthalene (Fig. 4). The presence of benzo(b)fluoranthene raises concern since it has been classified by the IARC and EPA as a probable human carcinogen. This water body is used for several human activities, such as fishing and salt extraction, by the people living around it, thereby exposing them to several dangers and the risk of getting cancer[10].

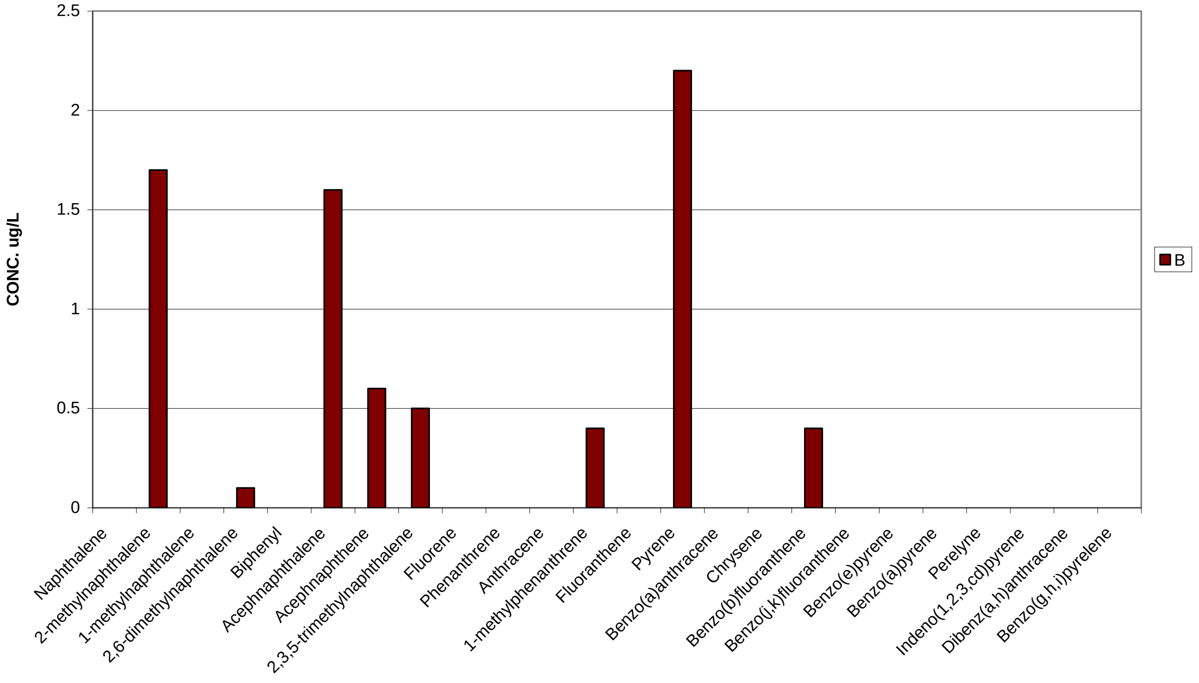

**FIGURE 4.** The average PAHs found in the Benya lagoon.

## Sakumono Lagoon

The Sakumono lagoon (D) receives effluents from an oil refinery and an aluminum facility. The following PAHs were determined: naphthalene, 2-methylnaphthalene, 2,6-dimethylnaphthalene, acephnaphthalene, anthracene, 1-methylphenanthrene, phenanthrene, fluoranthene, pyrene, acephnaphthene, 2,3,5-trimethylnaphthalene, fluorene, chrysene, and benzo(b)fluoranthene (Fig. 5). The presence of benzo(b)fluoranthene raises concern since it has been classified by the IARC and EPA as a probable human carcinogen. This water body is used for fishing by the people living around it, thereby exposing them to some dangers and the risk of getting cancer[10].

## Lower Volta Estuary

The lower Volta (E) empties Lake Volta, the largest manmade lake in the world that, via the Aksombo Dam, provides hydroelectric power to Ghana before the water reaches the estuary. There are quite a number of old electrical transformers, some in still use and others not in use. Most of these transformers have some organic chemicals as the dielectric material in them. It is therefore possible for some leakage

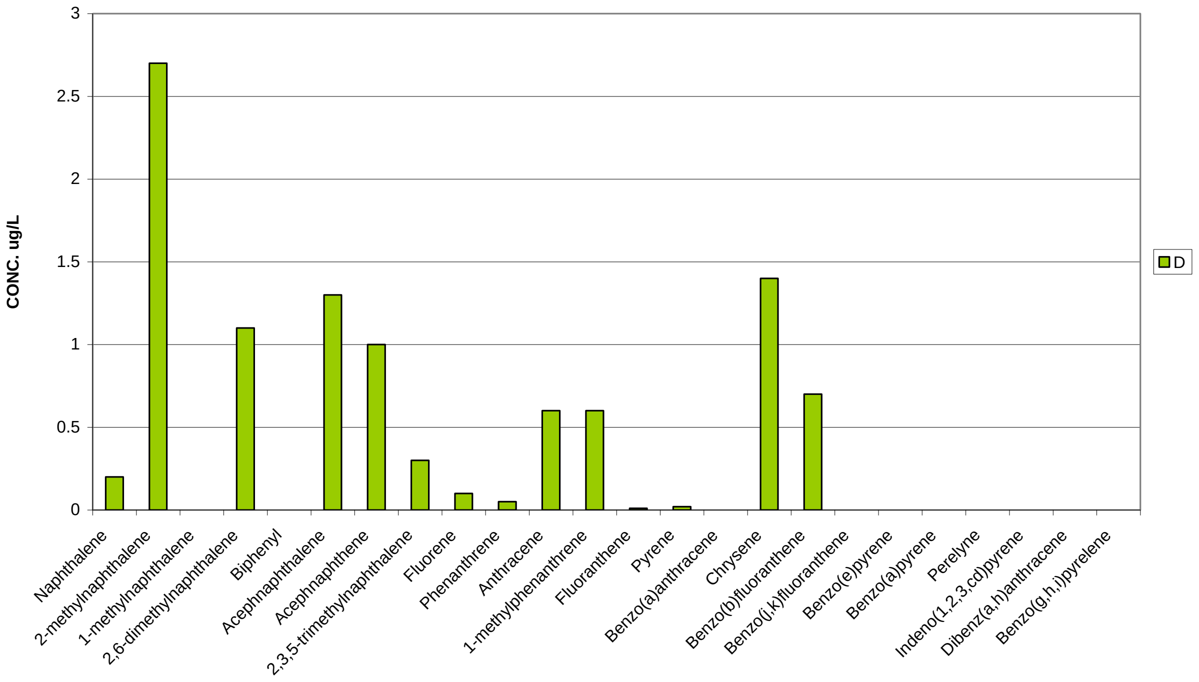

**FIGURE 5.** The average PAHs found in the Sakumono lagoon.

to the lake and that may have contributed to the high total PAHs in its estuary. The following PAHs were determined: naphthalene, 2-methylnaphthalene, 2,6-dimethylnaphthalene, biphenyl, acephnaphthalene, pyrene, 1-methylphenanthrene, acephnaphthene, 2,3,5-trimethylnaphthalene, fluorene, chrysene, and benzo(a)anthracene (Fig. 6). The presence of benzo(a)anthracene raises concern. This water body is also used for several human activities, including fishing and drinking, by the people living around it, thereby exposing them to some dangers and the risk of getting cancer[10].

## Keta Lagoon

The Keta lagoon, apart from the other activities that pertain in all the sites, has a number of onion farms around it and any chemical used on them may enter into the lagoon. The following PAHs were determined: naphthalene, 2-methylnaphthalene, 2,6-dimethylnaphthalene, biphenyl, acephnaphthalene, pyrene, acephnaphthene, 2,3,5-trimethylnaphthalene, fluorene, phenanthrene, chrysene, benzo(j,k)fluoranthene, fluoranthene, and 1-methylphenanthrene (Fig. 7). The presence of benzo(j,k)fluoranthene raises concern. This water body is used for fishing by the people living around it, thereby exposing them to some dangers and the risk of getting cancer[10].

## Narkwa Lagoon

There were high rates of indiscriminate dumping and burning of municipal waste at the Narkwa lagoon (C) vicinity. Coal tar is also used extensively to coat the canoes (fishing vessels) and most of the two-stroke engines of the outboard motors used are in poor condition, which may contribute to some PAH emissions. The following PAHs were determined: naphthalene, 2-methylnaphthalene, 2,6-dimethylnaphthalene, biphenyl, phenanthrene, 1-methylnaphthalene, pyrene, anthracene, acephnaphthalene, acephnaphthene, 2,3,5-trimethylnaphthalene, benzo(j,k)fluoranthene, and 1-methylphenanthrene (Fig. 8). The high level of

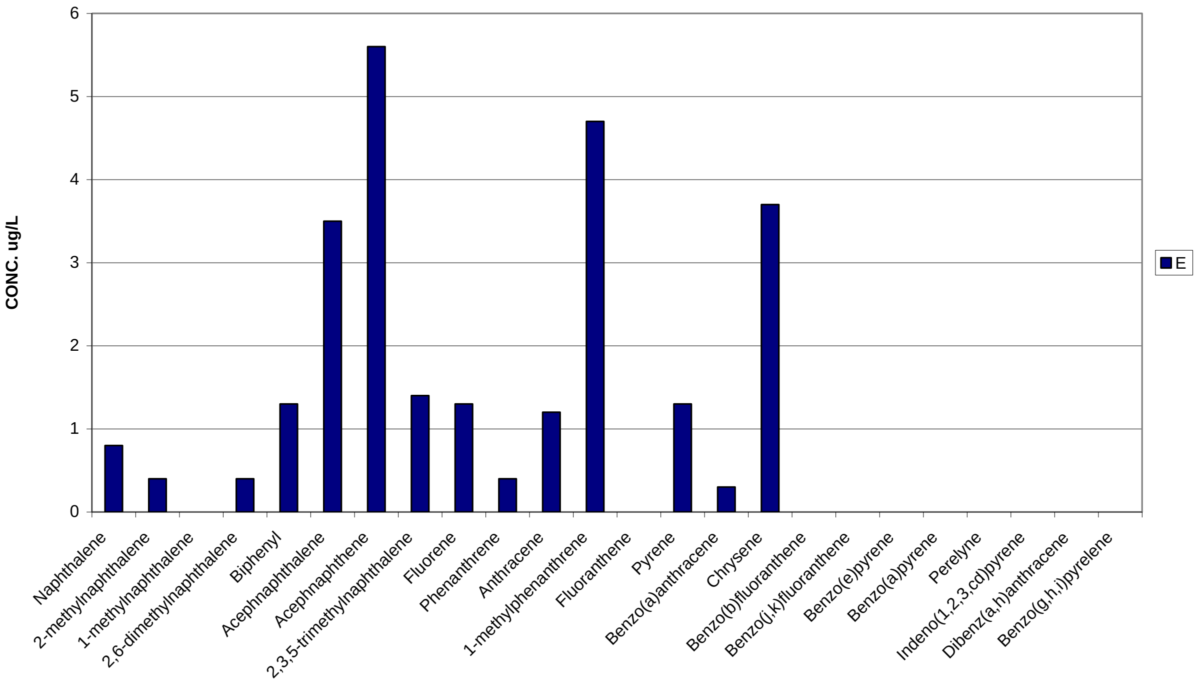

**FIGURE 6.** The average PAHs found in the lower Volta estuary.

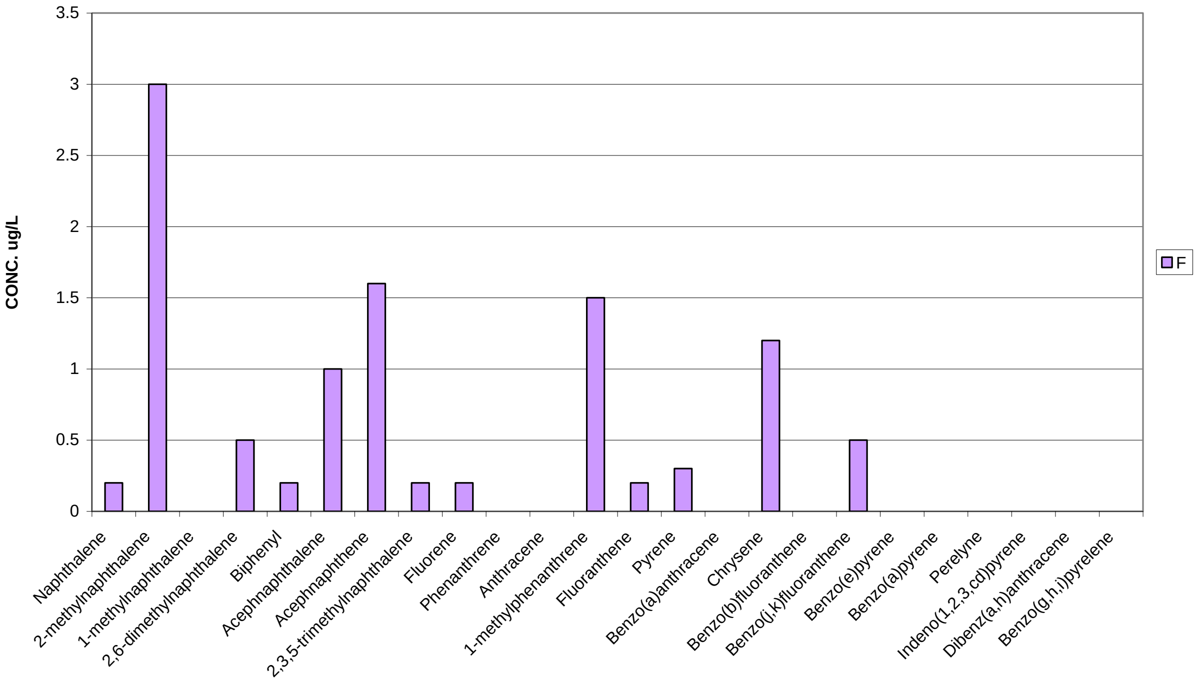

**FIGURE 7.** The average PAHs found in the Keta lagoon.

benzo(j,k)fluoranthene present in this water body raises concern. This water body is used for fishing activities by the people living around it, thereby exposing them to some dangers and the risk of getting cancer[10].

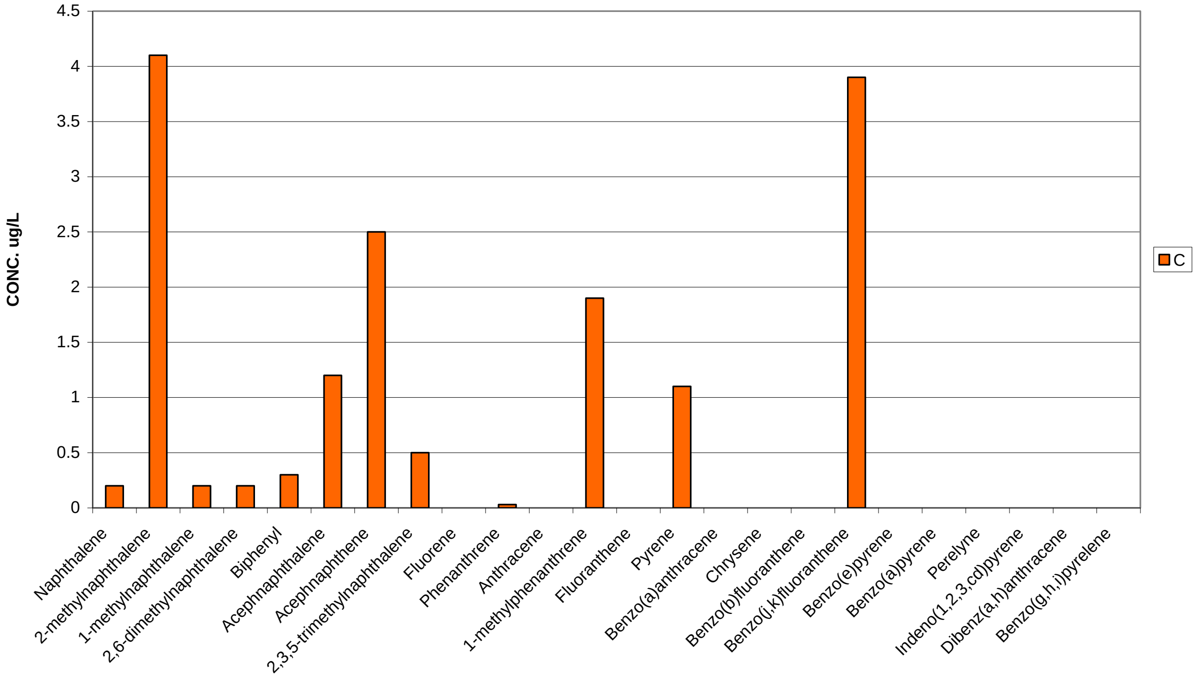

**FIGURE 8.** The average PAHs found in the Narkwa lagoon.

## Cancer Risk Assessment

A human health cancer risk assessment was carried out on the PAHs benzo(a)anthracene, benzo(b)fluoranthene, and benzo(j,k)fluoranthene (Table 2) using Human Health Evaluation computerized software-RISC 4.02[31], since their presence in these water bodies is of concern because they have been classified by the IARC and EPA as probable human carcinogens. These water bodies are used for fishing and for some domestic purposes by the people living around them, exposing them to several dangers and the risk of getting cancer[10]. Also, fish caught in these water bodies are sold to people in the urban areas and, as result, each one stands exposed.

The estimated lifetime cancer risk for adult users of the lower Volta estuary is as follows: (Oral): CTE = 6.5E-07 and RME = 2.0E-05, (Dermal); CTE = 9.7E-04 and RME = 7.3E-03 for chronic exposure to benzo(a)anthracene (Table 2). This means that between 7 out of 10,000,000 and 7 out of 1000 adults are likely to suffer cancer-related illness in their lifetime due to benzo(a)anthracene exposure. For children of similar exposure, between 5 out of 10,000,000 and 2 out of 1000 children (Table 2) are likely to suffer cancer-related illness in their lifetime due to benzo(a)anthracene exposure[37].

The estimated lifetime cancer risk for adult users of the Benya and Sakumono lagoons is between 8 out of 10,000,000 and 2 out of 100 adults (Table 2) that are likely to suffer cancer-related illness in their lifetime due to benzo(b)fluoranthene exposure. For children of similar exposure, between 6 out of 10,000,000 and 6 out of 1000 children (Table 2) are likely to suffer cancer-related illness in their lifetime due to benzo(b)fluoranthene exposure[37].

The estimated lifetime cancer risk for adult users of the Pra estuary and the Keta and Narkwa lagoons is between 10 out 100,000,000 and 1 out of 100 adults (Table 2) that are likely to suffer cancer-related illness in their lifetime due to benzo(j,k)fluoranthene exposure. For children of similar exposure, between 2 out of 100,000,000 and 3 out of 1000 children (Table 2) are likely to suffer cancer-related illness in their lifetime due to benzo(j,k)fluoranthene exposure[37].

**TABLE 2**
**The Result of the Human Health Cancer Risk Assessment**

| Sampling Point | Exposure Media | Exposure Route | CTE Adult | RME Adult | CTE Child | RME Child |
|---|---|---|---|---|---|---|
| Benz(a)anthracene | | | | | | |
| Lower Volta estuary | Water | Oral | 6.5E-07 | 2.0E-05 | 5.1E-07 | 1.8E-05 |
| | | Dermal | 9.7E-04 | 7.3E-03 | 2.8E-04 | 2.2E-03 |
| Benzo(b)fluoranthene | | | | | | |
| Benya lagoon | Water | Oral | 2.3E-05 | 7.6E-07 | 2.1E-05 | 5.9E-07 |
| | | Dermal | 1.3E-02 | 1.7E-03 | 3.7E-03 | 4.8E-04 |
| Sakumono lagoon | Water | Oral | 1.3E-06 | 3.8E-05 | 9.8E-07 | 3.5E-05 |
| | | Dermal | 2.8E-03 | 2.1E-02 | 8.0E-04 | 6.2E-03 |
| Benzo(j,k)fluoranthene | | | | | | |
| Pra estuary | Water | Oral | 1.9E-08 | 5.8E-07 | 1.5E-08 | 5.4E-07 |
| | | Dermal | 3.9E-05 | 2.9E-04 | 1.1E-05 | 8.7E-05 |
| Keta lagoon | Water | Oral | 9.7E-08 | 2.9E-06 | 7.5E-08 | 2.7E-06 |
| | | Dermal | 2.0E-04 | 1.5E-03 | 5.6E-05 | 4.3E-04 |
| Narkwa lagoon | Water | Oral | 7.5E-07 | 2.2E-05 | 5.8E-07 | 2.1E-05 |
| | | Dermal | 1.5E-03 | 1.1E-02 | 4.4E-04 | 3.4E-03 |

CTE = central tendency exposure, RME = reasonable maximum exposure.

## CONCLUSION

PAHs were found at all the sampling sites studied. Especially the lower-molecular-weight and the methylated PAHs, which are less carcinogenic to humans, were found at all sites. However, the presence of benzo(a)anthracene, benzo(b)fluoranthene, and benzo(j,k)fluoranthene is of much concern since their presence in these water bodies poses a greater health risk for the inhabitants. The human health cancer risk assessment studies carried out indicate that there were varied (possible) cancer risks among the inhabitants, depending on which water body they were in contact with[7].

There are various sources of PAHs that get into these water bodies. These may include (a) deposition of airborne particulates; (b) surface run-off from roads and land surfaces; (c) direct inputs from industrial and sewage effluents, and fossil fuel products; or (d) burning of solid waste. It is therefore important that the inhabitants, who know virtually nothing about PAHs and their harmful effects, are educated on the best practices to reduce indiscriminate burning of garbage (solid waste) to minimize PAH transport in the environment, and to take other actions against the dispersal of PAHs.

## ACKNOWLEDGMENT

The researcher would want to thank the Royal Society of Chemistry, U.K. for their financial support, which enabled us to get the necessary standards for this work. Also, I would want to thank the laboratory staff of the Chemistry Department, University of Cape Coast and (CSIR) Water Research Institute for their support.

## References

1. Dai, J., Li, S., Zhang, Y., Wang, R., and Yu, Y. (2008) Distributions, sources and risk assessment of polycyclic aromatic hydrocarbons (PAHs) in topsoil at Ji'nan city, China. *Environ. Monit. Assess*. **147,** 317–326.

2. Sarazin, L., Diana, C., Wafo, E., Pichard-Legadec, V., Schembri, T., and Monod, J.L. (2006) Determination of polycyclic aromatic hydrocarbons (PAHs) in marine, brackish, and river sediments by HPLC, following ultrasonic extraction. *J. Liq. Chromatogr. Relat. Technol.* **29(1),** 69–85.
3. Pearlman, R.S., Yalkowsky, S.H., and Banerjee, S. (1984) Water solubilities of polynuclear aromatic and heteroaromatic compounds. *J. Phys. Chem. Ref. Data* **13(2),** 555–562.
4. WHO (1987) Polynuclear aromatic hydrocarbons (PAH). In Air Quality Guidelines for Europe. World Health Organization Regional Office for Europe, Copenhagen. pp. 105–117.
5. Agency for Toxic Substances and Diseases Registry (1994) Toxicological Profile for Polycyclic Aromatic Hydrocarbons (PAHs): Update. U.S. Department of Health and Human Services, Public Health Services, Atlanta, GA.
6. Anyakora, C.A., Ogbeche, K.A., Palmer, P., Coker, H., Ukpo, G., and Ogah, C. (2004) A screen for benzo(a)pyrene, a carcinogen, in the water samples from the Niger Delta region. *Nig. J. Hosp. Med.* **14,** 288–293.
7. Gray, J., Ed. (2008) *State of the Evidence. The Connection between Breast Cancer and the Environment*. 5th ed. Breast Cancer Fund, San Francisco. pp. 1–127.
8. Baird, C. (1995) *Environmental Chemistry.* W.H. Freeman, New York. pp. 65–74.
9. Aynankora, C.A., Ogbeche, K.A., Palmer, P., Coker, H., and Ukpo, G. (2005) Analysis of polynuclear aromatic hydrocarbons in sediment samples of Niger delta region. *Chemosphere* **60,** 990–997.
10. Agency for Toxic Substances and Diseases Registry (ATSDR) (1995) Toxicological Profile for Polycyclic Aromatic Hydrocarbons (PAHs). U.S. Department of Health and Human Services, Public Health Services, Atlanta, GA.
11. Arias-Estevez, M. (2007) *Sorption of PAHs to Colloid Dispersion of Humic Substance in Water*. 4th ed. McGraw Hill Higher Education, New York. p. 315.
12. Abrajano, T.A. and Bopp, R.F. (1995) Sources and Chronology of Polycyclic Aromatic Hydrocarbon Deposition in Sediments from the Lower Hudson Watershed. http://www.rpi.edu/~abrajt/Goldschmidt.PDF
13. Intergovernmental Oceanographic Commission (IOC) (1998) Regional Workshop for Member States of Western Africa-GODAR-VI (Global Oceanographic Data Archaeology and Rescue Project), Accra Ghana, April 22–25, 1997. Organized by UNESCO. Workshop Report No. 136, Paris.
14. Kwesi, E. (1977) Biological, chemical and hydrological characters of coastal lagoons of Ghana, West Africa. *Hydrobiology* **56,** 157–174.
15. Biney, C.A. (1990) A review of some characteristics of freshwater and coastal ecosystems in Ghana. *Hydrobiology* **208,** 45–53.
16. Zakaria, M.P., Geik, K.H., Lee, W.Y., and Hayet, R. (2005) Landfill leachate as a source of polycyclic aromatic hydrocarbon (PAHs) to Malaysian waters. *Coastal Mar. Sci.* **29(2),** 116–123.
17. Essumang, D.K., Dodoo, D.K., Obiri, S., and Oduro, A.K. (2006) Analysis of polycyclic aromatic hydrocarbons in soil dust in the Kumasi Metropolis of Ghana, *J. Environ. Monit. Assess.* **121,** 401–408.
18. Canadian Council of Ministers of the Environment (CCREM) (1999) Canadian Water Quality Guidelines. Prepared by Task Force on Water Quality Guidelines.
19. Karthikeyan, R., Kulakov, P., Leven, B., Pierzynski, G., and Erickson, L.E. (2000) Vegetated treatment of vehicle wash sediments: a field demonstration. Proceedings of the 2000 Conference on Hazardous Waste Research, Denver, May 23–25. pp. 159–166.
20. Gilbert, E., Dodoo, D.K., Okai-Sam, F., Essumang, D.K., and Quagraine, E.K. (2006) Characterization and source assessment of heavy metals and polycyclic aromatic hydrocarbons (PAHs) in sediments of the Fosu Lagoon, Ghana. *J. Environ. Sci. Health A Tox. Hazard. Subst. Environ. Eng.* **41(12),** 2747–2775.
21. Essumang, D.K., Adokoh, C.K., Afriyie, J., and Mensah, E. (2009) Source assessment and analysis of polycyclic aromatic hydrocarbon (PAH's) in the Oblogo waste disposal sites and some water bodies in and around the Accra Metropolis of Ghana. *J. Water Resour. Protect*. **1(6),** 456–468.
22. Davis, C.S., Fellin, P., and Otson, R. (1987) A review of sampling method for polyaromatic hydrocarbon in air. *J. Air Pollut. Control Assoc.* **37,** 1397–1408.
23. Edwards, C.A. (2002) The Fate of Outboard Engine Combustion Products in the Mary River Floodplain Billabongs. Are They a Pollutant? Report 08/2002. Northern Territory Department of Infrastructure, Planning and Environment, Darwin, Australia.
24. Kelly, C.A., Ayoko, G.A., Brown, R.J., and Swaroop, C.R. (2005) Underwater emissions from a two-stroke outboard engine: a comparison between an EAL and an equivalent mineral lubricant. *Mater. Des*. **26(7),** 609–617.
25. Boström, C., Gerde, P., Hanberg, A., Jernström, B., Johansson, C., Kyrklund, T., Rannug, A., Törnqvist, M., Victorin, K., and Westerholm, R. (2002) Cancer risk assessment, indicators, and guidelines for polycyclic aromatic hydrocarbons in the ambient air. *Environ. Health Perspect.* **110(Suppl 3),** 451–489.
26. Maskaoui, K., Zheng, T., Hong, H., Yu, Z., Yuan, J., and Hu, Z. (2004) Preliminary study on PAH degradation by bacteria from contaminated sediments in Xiamen Western Sea, Fujian, China. *Chin. J. Oceanol. Limnol.* **22(4),** 431–435.
27. Alonge, D.O. (1988) Carcinogenic polycyclic aromatic hydrocarbon (PAH) determined in Nigerian Kundi (smoked dried meat). *J. Sci. Food Agric*. **43,** 167–173.
28. Douglass, F. (2004) *GC/MS Analytical Methods*. 2nd ed. Academic Press, New York. pp. 112–115.
29. Edgell, K. and Wesselman, R. (1989) USEPA Method Study 36 - Sw-846 Methods 8270/3510 GC/MS Method for Semivolatile Organics: Capillary Column Technique Separatory Funnel Liquid-Liquid Extraction. U.S. Environmental Protection Agency, Washington, D.C. EPA/600/4-89/010 (NTIS Pb89190581).

30. US EPA (1989a) Evaluation of Sample Extract Cleanup Using Solid-Phase Extraction Cartridges. Project Report, December 1989.
31. US EPA (1989b) Risk Assessment Guidance for Superfund (RAGS). Vol. 1. Human Health Evaluation Manual (Part A). Office of Emergency and Remedial Response, Washington, D. C. OWSER Directive 9285. 7 – 01A. EPA/540/1-89/002.
32. Maskaoui, K., Zhou, J.L., Hong, H.S., and Zhang, Z.L. (2002) Contamination by polycyclic aromatic hydrocarbons in the Jiulong River Estuary and Western Xiamen Sea, China. *Environ. Pollut.* **118(1),** 109–122.
33. Zhou, J.L. and Maskaoui, K. (2003) Distribution of polycyclic aromatic hydrocarbons in water and surface sediments from Daya Bay, China. *Environ. Pollut.* **121,** 269–281.
34. Wattayakorn, G. (2003) Polycyclic aromatic hydrocarbons in the Chao Phraya Estuary, Thailand. *J. Sci. Res. Chula. Univ.* **28(Special Issue I [NRC-EHWM]),** 15–27.
35. El-Deeb, M.K.Z. and Emara, H.I. (2005) Polycyclic aromatic hydrocarbons and aromatic plasticiser materials in the seawater of Alexandria coastal area. *Egypt. J. Aquat. Res*. **31(Special Issue),** 15–24.
36. Simpson, C.D., Mosi, A.A., Cullen, W.R., and Reimer, K.J. (1996) Composition and distribution of polycyclic aromatic hydrocarbon contamination in surficial marine sediments from Kitimat harbour. *Can. Sci. Total Environ*. **181,** 265–278.
37. US EPA (1995) Superfund Record of Decision: Record of Decision Harris Corporation/Palm Bay Facility Site. USA Directive EPA/ROD/R04-95/211. FLD000602334.
